# Cytochalasin B-induced membrane vesicles convey angiogenic activity of parental cells

**DOI:** 10.18632/oncotarget.19723

**Published:** 2017-07-31

**Authors:** Marina O. Gomzikova, Margarita N. Zhuravleva, Regina R. Miftakhova, Svetlana S. Arkhipova, Vladimir G. Evtugin, Svetlana F. Khaiboullina, Andrey P. Kiyasov, Jenny L. Persson, Nigel P. Mongan, Richard G. Pestell, Albert A. Rizvanov

**Affiliations:** ^1^ Kazan Federal University, Kazan, 420008, Russia; ^2^ Department of Microbiology and Immunology, University of Nevada, Reno, Nevada, 89557, USA; ^3^ Department of Translational Medicine, Lund University, 205 02 Malmö, and Department of Molecular Biology, 901 87 Umeå, Umeå University; ^4^ Cancer Biology and Translational Research, School of Veterinary Medicine and Science, University of Nottingham, LE12 5RD, UK; ^5^ Department of Pharmacology, Weill Cornell Medicine, 1300 York Ave., New York, NY, 10065, USA; ^6^ Pennsylvania Cancer and Regenerative Medicine Research Center, Baruch S. Blumberg Institute, Pennsylvania Biotechnology Center, 100 East Lancaster Avenue, Suite, 222, Wynnewood, PA 19096. USA; ^7^ Lee Kong Chian School of Medicine, Nanyang Technological University, Singapore 637551, Singapore

**Keywords:** extracellular vesicles, membrane vesicles, cytochalasin B-induced membrane vesicles, angiogenesis, cell-free therapy

## Abstract

Naturally occurring extracellular vesicles (EVs) play essential roles in intracellular communication and delivery of bioactive molecules. Therefore it has been suggested that EVs could be used for delivery of therapeutics. However, to date the therapeutic application of EVs has been limited by number of factors, including limited yield and full understanding of their biological activities. To address these issues, we analyzed the morphology, molecular composition, fusion capacity and biological activity of Cytochalasin B-induced membrane vesicles (CIMVs). The size of these vesicles was comparable to that of naturally occurring EVs. In addition, we have shown that CIMVs from human SH-SY5Y cells contain elevated levels of VEGF as compared to the parental cells, and stimulate angiogenesis *in vitro* and *in vivo*.

## INTRODUCTION

Cell therapy has been proven as an effective method for injury treatment [1-4]. Although considerable advances were made, current understanding of the mechanisms of transplanted cell therapeutic effects is incomplete. It is known that the tissue repair achieved is independent of transplanted cell-engraftment [5-6] and it can be achieved by injection of conditioned media derived from stem cell cultures [7-8] suggesting the therapeutic effect is paracrine and associated with the release of soluble factors. According to the paracrine hypothesis, transplanted cells secrete biologically active factors including cytokines and growth factors into extracellular space activating resident cells and supporting tissue growth and repair [9-10]. More recent evidence also supports a paracrine role for extracellular vesicles (EVs) [11]. EV are important vehicles carrying proteins, mRNA, miRNAs and siRNA and provide a mechanism for cell crosstalk within tissues. Therefore, EVs could have great therapeutic potential as vehicles to deliver biologically active molecules. The therapeutic capacity of EVs was shown using models of a kidney injury [12], heart [13-14], liver [15] and nervous tissue injury [16].

In the last decade, technological advances have improved the yield and quality of isolated EVs [17-19]. Pick and colleagues [20] demonstrated that active agitation of Cytochalasin B-treated cells stimulated the production of membrane vesicles [20]. The membrane vesicles produced in this way retain normal cellular signaling capacities [20]. Mao and colleagues used the Cytochalasin B induced membrane vesicles as vectors for drug and nanoparticles delivery [21]. However the biological activities of membrane vesicles obtained from Cytochalasin B-treated cells have not been evaluated.

In this study, human neuroblastoma SH-SY5Y cells [22] were chosen as a source of CIMV due to their well-established angiogenic properties. It is known that neuroblastoma is a highly vascular solid tumor [23]. Previously we have shown that SH-SY5Y cells induce formation of capillary-like structures by human mesenchymal stem cells [24]. Therefore, in the current study we sought to determine whether Cytochalasin B induced membrane vesicles (CIMVs) derived from SH-SY5Y bone marrow neuroblastoma cells could promote angiogenesis. To this end we examined the morphology, molecular composition, fusion capacity and biological activity of membrane vesicles generated from Cytochalasin B-treated SH-SY5Y cells. We have found that CIMVs maintain angiogenic properties of the parental cells *in vitro* and *in vivo*.

## RESULTS

### Characterization of CIMVs

**Figure 1:**
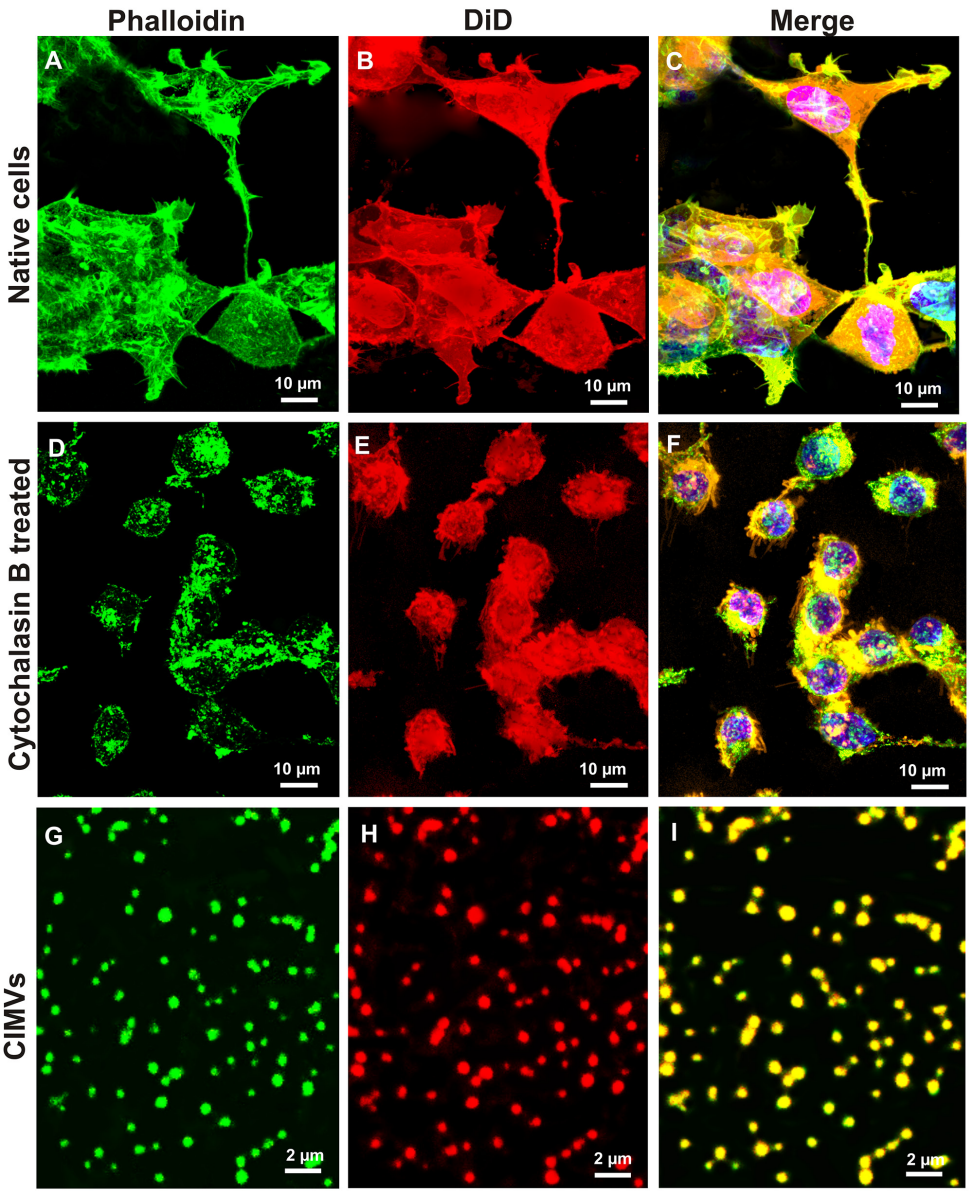
Comparison of the actin cytoskeleton structure in native and cytochalasin B treated cells and presence of actin filaments in CIMVs SH-SY5Y cells were pre-stained with the cytoplasmic membrane dye DiD, then native cell **(A-C)**, cells after 30 min incubation with cytochalasin B **(D-F)**, or CIMVs **(G-I)** were fixed and stained with Phalloidin-Alexa488. Micrographs indicate that cytochalasin B induces actin cytoskeleton disorganization and as a consequence the loss of cells form. The actin cytoskeleton fragments which were formed under the effect of cytochalasin B are part of the CIMVs (G-I).

Cytochalasin B targets actin cytoskeleton [25], therefore we first sought to determine the effect of Cytochalasin B treatment on actin fibers in SH-SY5Y cells as well as in CIMVs. Continuous actin microfilaments are found in untreated control SH-SY5Y cells (Figure 1). In contrast, in Cytochalasin B-treated SH-SY5Y cells actin microfilaments were disrupted and appeared as a sub-membrane “islands” (Figure 1). Confocal microscopy revealed actin cytoskeleton fragments in CIMVs (Figure 1).

**Figure 2:**
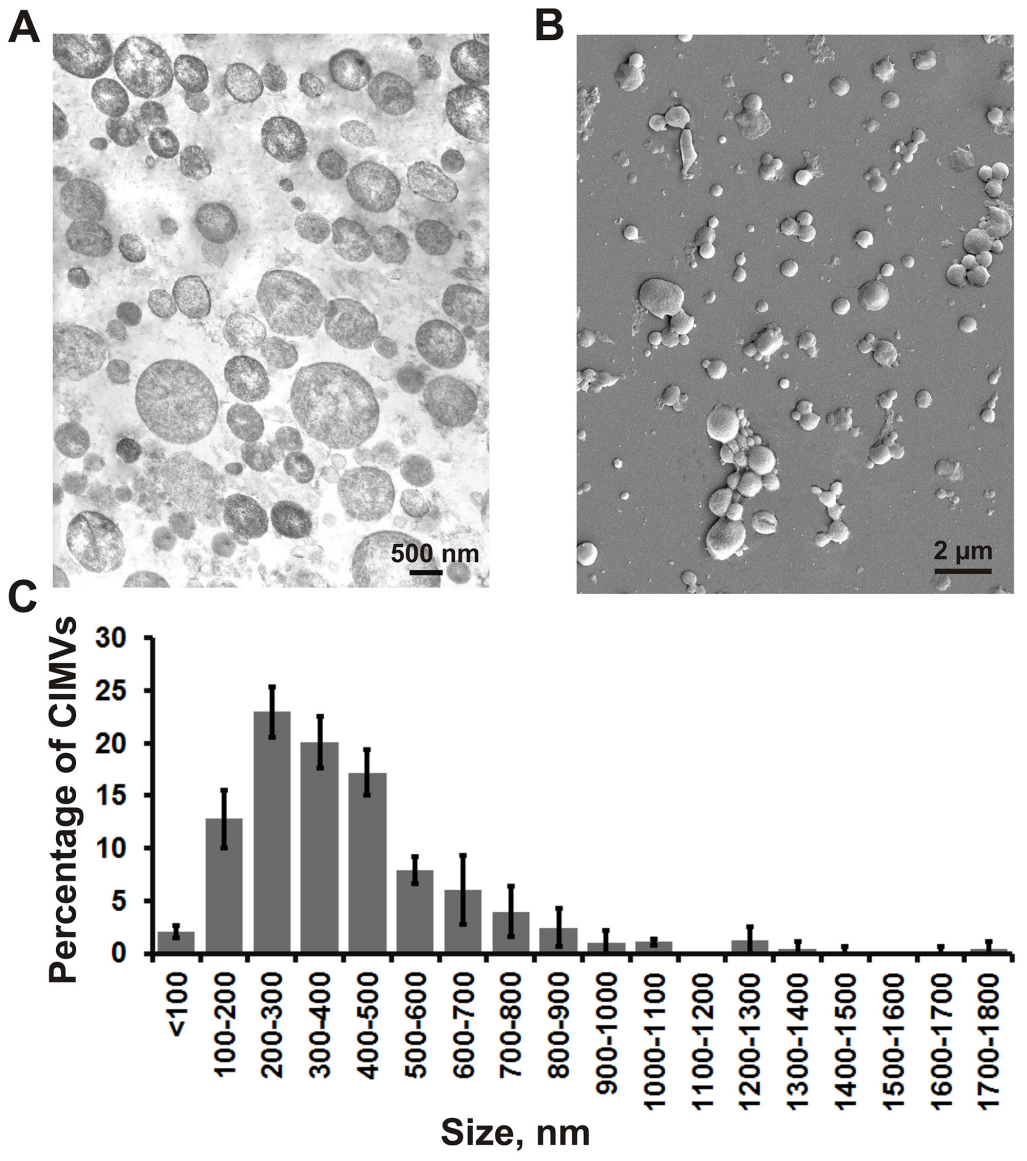
The structure, morphology and size distribution of CIMVs Transmission electron microscopy **(A)** and scanning electron microscopy **(B)** was used to analyze the biophysical properties of SH-SY5Y properties of CIMVs. **(C)** Size distribution of CIMVs SH-SY5Y. For statistical analysis, at least six electron microscope images were analyzed from three independent experiments.

Next, we investigated the structure and size of SH-SY5Y derived CIMVs using transmission and scanning electron microscopy. We found that CIMVs had a round-shaped structure with a diameter varying from 80 to 1790 nm. It should be noted that the majority of CIMVs (96%) had a diameter of 100-1000 nm (Figure 2C) which is within the range of naturally occurring EVs.

### Interaction of CIMVs with recipient SH-SY5Y cells

**Figure 3:**
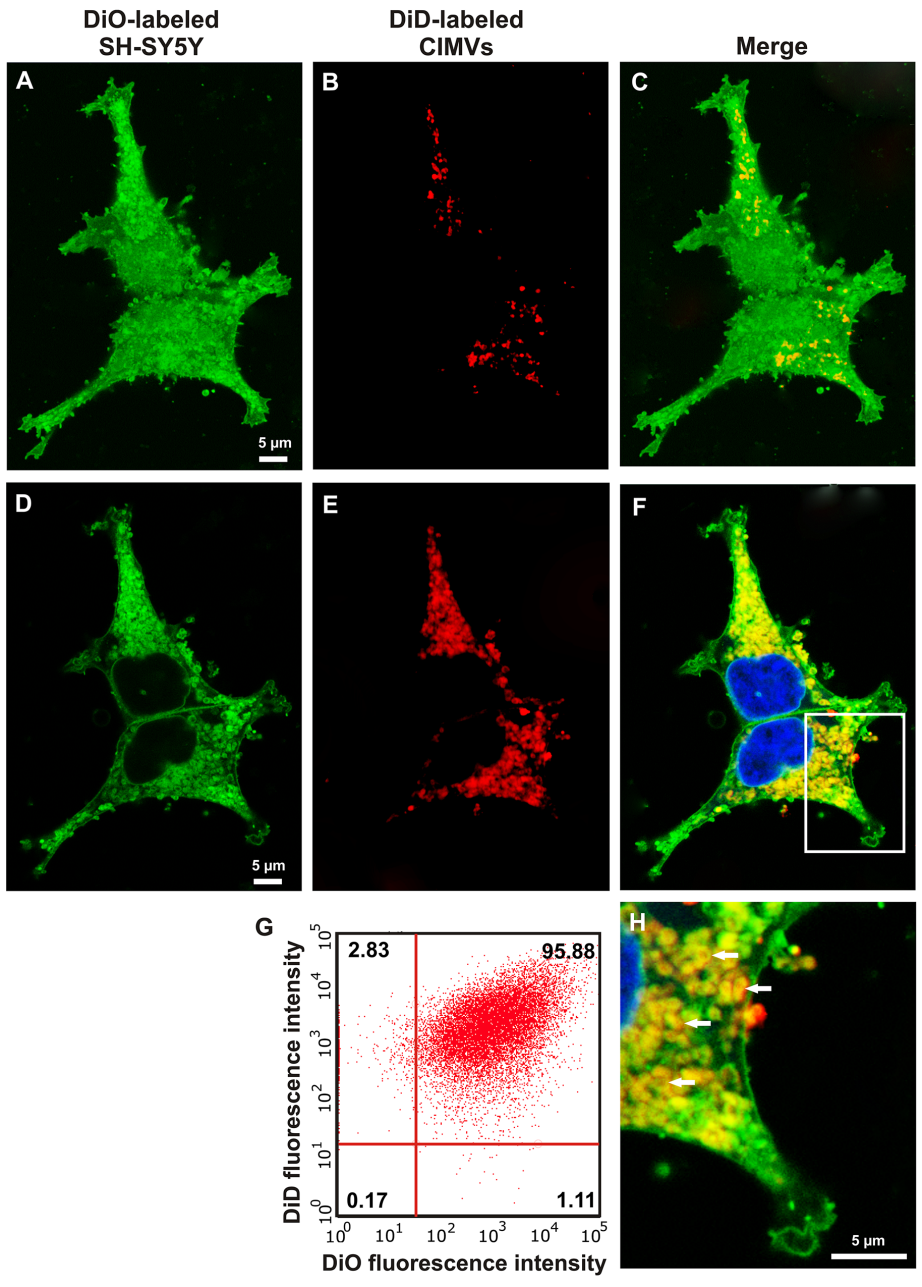
Interaction of CIMVs with recipient cells Fluorescent microscopy Z-stack **(A-F,H)** and flow cytometry analysis **(G)** of SH-SY5Y recipient cells 24h after 10 μg/ml CIMVs application were carried out. Recipient cells were pre-labeled with DiO cytoplasmic membrane dye and CIMVs were pre-labeled with DiD dye. Cells surface (A-C) and cells inner content (D-F,H) were analyzed for the presence of CIMVs membrane component.

The cytoplasmic membrane of CIMVs and recipient SH-SY5Y cells were differentially labeled with DiD (red fluorescent dye) and DiO (green fluorescent dye), respectively. DiD and DiO dyes have non-overlapping excitation/emission spectrum (DiD – 644/665 nm; DiO – 484/501 nm) and do not transfer between cytoplasmic membranes of neighboring cells [26]. CIMVs were incubated with the recipient SH-SY5Y cells and the surface and inner content of recipient SH-SY5Y cells were analyzed to determine the presence of the CIMVs membrane component. FACS analysis revealed that the majority of cells (95.88%) had DiO and DiD dual fluorescence (Figure 3G). Round structures with red fluorescence (DiD positive) were detected on SH-SY5Y surface (Figure 3A-3C). Round structures with dual red and green fluorescence were detected within the cytoplasm of recipient SH-SY5Y (Figure 3D-3F, 3H). This data suggests that CIMVs merge with SH-SY5Y membranes and engage in the endosome trafficking pathway (Figure 3G).

### CIMVs exhibit angiogenic activity *in vitro*

Previously we have shown that SH-SY5Y cells stimulate mesenchymal stem cell (MSC) formation of capillary-like tubes in matrigel [24]. Therefore we sought to compare whether SH-SY5Y derived CIMVs retain this pro-angiogenic property of the parental SH-SY5Y cells. SH-SY5Y cells were mitotically inactivated using mitomycin C to eliminate the changes in secreted growth factors concentration introduced by active cell proliferation.

**Figure 4:**
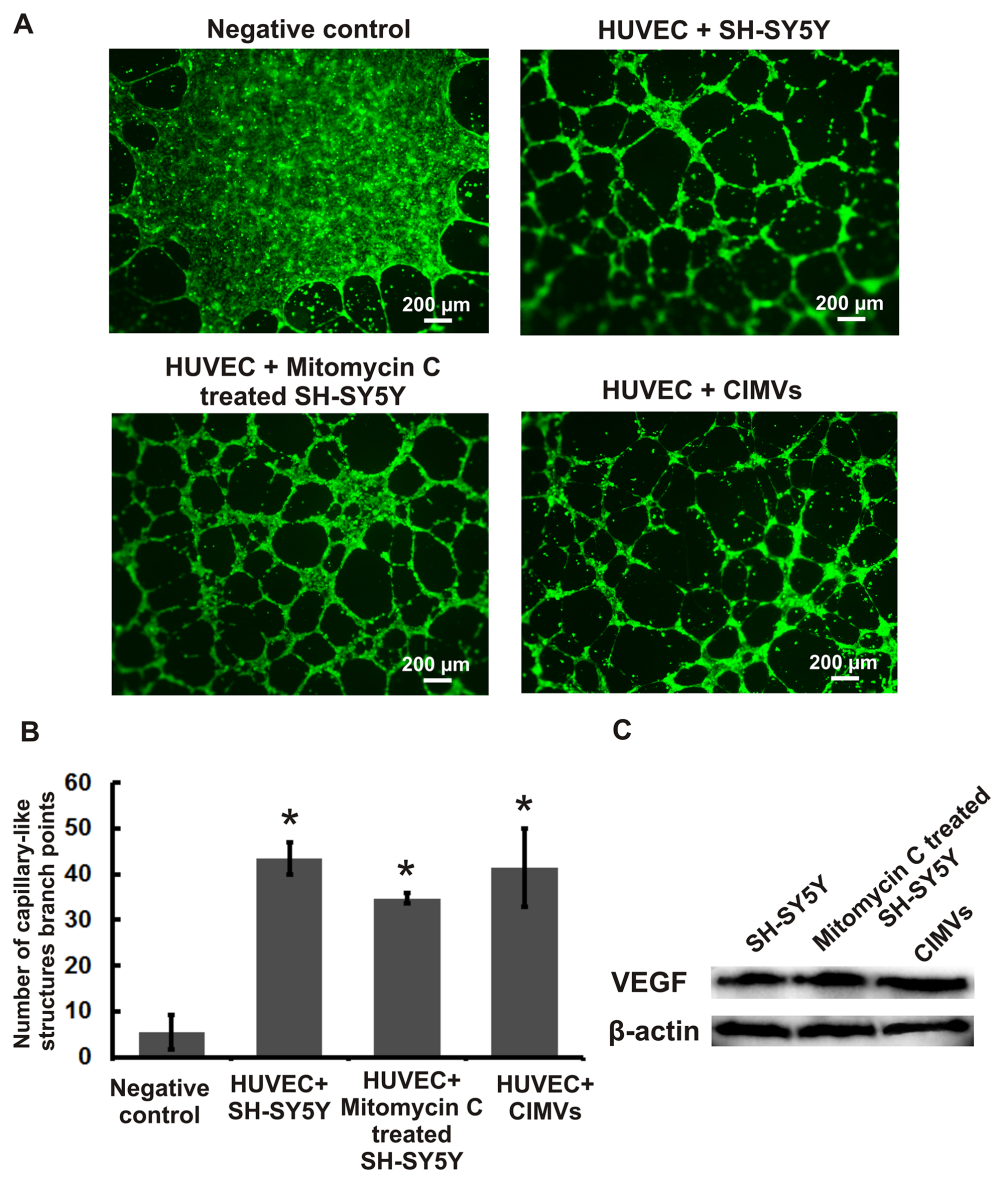
Evaluation of *in vitro* pro-angiogenic potential of mitotically inactivated cells and CIMVs SH-SY5Y **(A)** Fluorescence micrographs of HUVEC culture after 16 hours of incubation with native/ mitotically inactivated SH-SY5Y cells or CIMVs. **(B)** Quantitation of the capillary-like structure branch points number formed by HUVECs after 16 hours of co-culture with native/ mitotically inactivated SH-SY5Y cells or CIMVs. The data represents mean {plus minus} SD. For statistical analysis six well of 96-well plate were analyzed per experimental group. **(C)** Representative western blots showing VEGF and β-actin in native/ mitotically inactivated SH-SY5Y cells or CIMVs.

HUVECs capillary-like tube formation in matrigel was examined *in vitro* to determine the pro-angiogenic properties of CIMVs. HUVECs were pre-stained with CFDA-SE (eBioscience, USA) and co-cultured with CIMVs or mitotically inactivated SH-SY5Y cells for 16 hours before analysis of the capillary-like network (Figure 4A). The mean number of HUVECs capillary-like network branch points was calculated using Image J software. The mean number of capillary-like network branch points in the negative control group (HUVECs cultured in MCDB131 medium with 1% FBS) was 5.3 ± 3.8 (Figure 4B). As expected, HUVECs cultured with SH-SY5Y cells showed a significantly increased number of capillary-like structures (43.5 ± 3.5; p<0.01) as compared to negative control. Furthermore, co-culture of HUVECs with mitotically inactivated SH-SY5Y cells also induced a significantly increased number of capillary-like structures (34.7 ± 1.15; p<0.01). Consistent with our hypothesis that CIMVs retain the pro-angiogenic activity of the parental SH-SY5Y cells, CIMVs were shown to also induced a significant increase in capillary-like structures as compared to the negative control group (41.3 ± 8.5;p<0.01) (Figure 4B). Indeed, under the conditions employed we found that CIMVs induced comparable HUVECs capillary-like structures as achieved by mitotically competent SH-SY5Y *in vitro.* Furthermore, CIMV induced angiogenesis was not associated with changes in HUVECs viability (Figure 3, Supplementary Data).

Endothelial cell proliferation is regulated primarily by ligands for receptor tyrosine kinases (RTKs) [23]. Therefore we sought to determine whether the angiogenic activity of CIMVs is associated with VEGF. We found ∼1.5 fold higher level of VEGF in SH-SY5Y-derived CIMVs as compared to SH-SY5Y cells (Figure 4C).

### CIMVs stimulate angiogenesis *in vivo*

**Figure 5:**
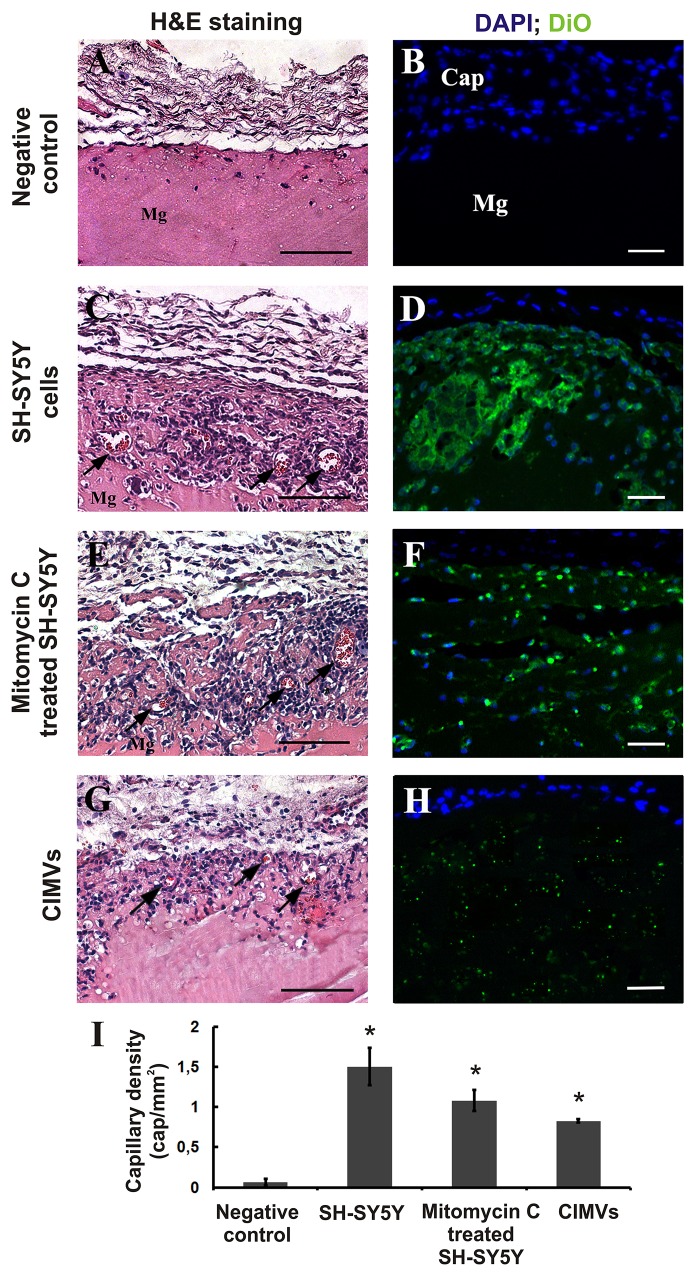
CIMVs stimulate angiogenesis *in vivo* Representative images of control matrigel plugs **(A,B)**, matrigel plugs with native **(C, D)**, mitotically inactivated **(E, F)** SH-SY5Y cells or CIMVs **(G, H)** 8 days after subcutaneous injection in Rattus norvegicus (6 animals per experimental group). Hematoxylin/eosin staining and fluorescence micrographs are shown. Mg - Matrigel, Cap - fibrous capsule. Arrows mark the position of the sprouting blood capillaries. **(I)** Quantitation of the capillary density in matrigel plugs. The data represents mean {plus minus} SD. For statistical analysis, fifteen hematoxylin and eosin stained slides per animal were analyzed.

Given the increased capillary-like structure formation by HUVECs incubated with CIMVs *in vitro*, we next examined the pro-angiogenesis activity of CIMV *in vivo* using the matrigel plug angiogenesis assay in rat model. CIMVs and cells were first stained with DiO, then matrigel solutions (200 μl) containing either CIMVs or native or mitotically inactivated SH-SY5Y cells were prepared and injected subcutaneously into rat abdominal flanks. Histological examination of matrigel implants was conducted 8 days later. Native, mitotically inactivated SH-SY5Y cells and CIMVs were detected by confocal microscopy in the matrigel implants 8 days after injection (Figure 5D, 5F, 5H). Also, newly developed blood capillaries were observed in matrigel containing native, mitotically inactivated SH-SY5Y cells and CIMVs (Figure 5C, 5E, 5G). The number of blood vessels in control matrigel (without cells or CIMVs) was 0.065±0.04 cap/mm^2^. More blood vessels were found in matrigel containing native SH-SY5Y cells, with an average capillary density 23-fold higher (1.5±0.23 cap/mm^2^; p<0.01) than that in control (Figure 5I). Similar to native cells, the number of the new capillaries was 16.6 fold higher in matrigel containing mitotically inactivated SH-SY5Y cells (1.08±0.1 cap/mm^2^; p<0.01) than that in control. Finally, matrigel implants containing CIMVs achieved 12.7 fold more capillary density (0.83±0.02 cap/mm^2^; p<0.01) as compared to untreated control. We conclude that the SH-SY5Y derived CIMVs retain the pro-angiogenic properties of the parental cells. Interestingly, the angiogenic property of CIMVs was lower than that of SH-SY5Y parental cells, but closely resembling that of mitotically inactive cells. Although this may indicate that SH-SY5Y retains additional pro-angiogenic activity as compared to CIMVs, CIMVs also induced a statistically significant increase in angiogenesis as compared to control. As outlined later, the therapeutic use of CIMVs, but not native cells, is mechanistically feasible.

## DISCUSSION

EVs are important vehicles carrying biologically active molecules providing cell cross talk within the tissue [27]. Recently there has been significant interest in the potential application of EVs as tools for cell-free delivery of therapeutics as unlike parental cells, it is believed there is no risk of tumor formation [28]. However, to date the yield of the naturally occurring EVs has been too low and is insufficient for therapeutic applications. Therefore, the development of new methods to generate EVs has been an area of active research. The use of cytochalasin B is now a well-established approach to generate sufficient quantities of membrane vesicles for therapeutic development [20]. However, it remained unclear whether CIMVs retain cell regulatory capacity similar to those of naturally occurring EVs.

Studies of the molecular mechanisms regulating extracellular vesicles formation, revealed that budding of microvesicles requires local actin cytoskeleton disorganization by the Ca^2+^-dependent protease, calpain, where digested actin microfilament fragments become captured within the microvesicles [29-30]. Since cytochalasin B mechanisms of action are similar to those of calpain, the presence of actin microfilaments within the CIMVs was investigated. Here we found that actin molecules are captured in CIMVs. Furthermore, we show that CMIVs were ∼100-1000 nm in diameter, which is comparable with naturally occurring EVs [31-35].

Naturally occurring EVs can acquire and transport biologically active molecules. Also, EVs are able to fuse directly with the cytoplasmic membrane of recipient cells and be captured by endocytosis [36]. However whether the membrane fusion capacity and mechanisms of intracellular transport were retained by CIMVs was unknown. Here for the first time we have shown that CIMVs can fuse with the recipient cell membrane. Also, we found that CIMVs, like naturally occurring EVs, can be transported via endocytosis. This data suggests that CIMVs may have similar bio-physical properties, closely resembling that of natural microvesicles, and thus are likely suitable as vectors for delivery of bioactive molecules.

Naturally occurring EVs can deliver biologically active substances to recipient cells, and thereby regulate cell growth and proliferation [37-38]. Therefore, we investigated the effect of CIMVs on HUVECs capillary-like structures formation *in vitro*. Our data, for the first time indicates that CIMVs can induce the angiogenesis, similar to that previously described for the parental cells [24]. We believe that VEGF could, in part, explain the pro-angiogenic properties of CIMVs.

The pro-angiogenic potential of the SH-SY5Y neuroblastoma cells derived CIMVs was also confirmed using an *in vivo* model. Tumor cells secrete various cytokines and chemokines attracting endothelial cells [39] and thereby stimulate angiogenesis. Subcutaneously injected in matrigel cells or CIMVs of SH-SY5Y induced the blood capillary sprouting by activation pro-inflammatory and pro-angiogenic pathways [39]. Although the angiogenic activity of native SH-SY5Y cells was 1.8 times higher than CIMVs, it was still comparable to that of mitotically inactive cells. We believe that the high angiogenic activity of SH-SY5Y cells was due to the ability of these cells to proliferate, thus continuously increasing the number of tumor cells releasing growth factors. On the contrary, CIMVs and mitomycin C treated cells fail to proliferate, thus although they able to secrete bioactive molecules, the amount of cytokines produced remains unchanged. The potential clinical advantages of this feature of CIMVs in terms of controlling therapeutic concentrations and minimizing off-target effects are clear.

In conclusion, we demonstrated that cytochalasin B-induced SH-SY5Y-derived membrane vesicles retain the properties of the donor cells, as they can stimulate angiogenesis *in vitro* and *in vivo*. Unlike the naturally occurring EVs, CIMVs can be produced in large quantity and be scaled to an industrial production level. This makes the CIMVs derived from primary cells (mesenchymal stem cells, endothelial cells, endothelial progenitor cells etc.) an attractive therapeutic approach to trigger the angiogenesis in clinical case of limb ischemia, diabetes-caused ischemic cardiovascular disease, ischemic stroke, myocardial infarction [40-42]. We propose that the CIMVs may serve as a tool for cell-free therapeutic biomolecules delivery.

## MATERIALS AND METHODS

### Cell culture

SH-SY5Y cells (ATCC® CRL-2266, American Type Culture Collection, Manassas, VA) were maintained in Dulbecco’s modified Eagle’s medium (DMEM; PanEco, Russia) with 10% fetal bovine serum (FBS; GE Healthcare, USA) and 2mM L-glutamine (PanEco, Russia) at 37°С with 5% CO_2_. Human umbilical vein endothelial cells (HUVECs) were isolated as described previously [43]. Experiments using human samples were reviewed and approved by the local Ethical Committee of Kazan (Volga region) Federal University based on article 20 of the Federal Legislation on “Health Protection of Citizens of the Russian Federation” № 323-FL, 21.11.2011. Informed consent was obtained from all patients for the use of specimens in research and the Helsinki Declaration observed. Briefly, the umbilical cord was washed with Hanks’ solution (PanEco, Russia) containing 100 I.U./mL penicillin, 100 μg/mL streptomycin (PanEco, Russia). Then, 1 cm of tissue was removed from each end of the cord. A catheter (Apexmed International, Netherlands) was inserted into the umbilical vein, the vein perfused with Hanks’ solution containing antibiotics. Umbilical vein endothelial cells were then dissociated by incubating with trypsin-EDTA (0.25%) solution (Life Technologies, USA) for 20 minutes at 37°С. Cells were retrieved by centrifugation (1500 rpm for 10 min), resuspended in HUVEC culture medium and incubated at 37°C in a humidified atmosphere containing 5% CO_2_. HUVECs phenotype was confirmed by flow cytometry (CD31^+^, CD105^+^, CD146^+^, CD144^+^, CD45^-^, CD14^-^). HUVECs were maintained in MCDB131 medium (Sigma-Aldrich, USA) with 20% FBS (GE Healthcare, USA) supplemented with non-essential amino acids (Life Technologies, USA), 10 ng/ml FGF2 (Sigma-Aldrich, USA), 10 ng/ml VEGF (Sigma-Aldrich, USA), 10 ng/ml insulin-like growth factor (Sigma-Aldrich, USA), 50 ng/ml endothelial cell growth supplement (Sigma-Aldrich, USA) and 10 ug/ml heparin (Sigma-Aldrich, USA).

### CIMVs production

CIMVs were prepared as described by Pick et al. [20], with modifications. Briefly, SH-SY5Y cells were washed twice with PBS, and incubated in DMEM containing 10 μg/ml of Cytochalasin B (Sigma-Aldrich, USA) for 30 min (37°C, 5% CO_2_). At the end of incubation, the cell suspension was vortexed vigorously for 30 sec and pelleted (100 g for 10 min). The supernatant was subject to two subsequent centrifugation steps (100 g for 20 min and 2000 g for 25 min). The resulting pellet contained CIMVs.

### Actin microfilaments and cytoplasmic membrane staining

SH-SY5Y cells and CIMVs were fixed (10% formalin for 15 min), washed twice with PBS and permeabilized using 0.1% Tween 20 for 15 min. Phalloidin conjugated with Alexa 488 (SantaCruz, USA) was added for 15 min to visualize actin. Nuclei were stained with Hoechst 33342 (SantaCruz, USA) for 10 min. Lipophilic dyes DiD and DiO (Life Technologies, USA) were used to visualize cell membranes. Cell suspension (1x10^6^ cells/ml) was incubated with 5μM of DiO or DiD dyes for 15 min (37°C, 5% CO_2_) and washed (3x) with complete medium (DMEM with 10% FBS, 2mM L-glutamine). Cells were analyzed by confocal laser scanning microscopy (Carl Zeiss LSM 780, Germany) and flow cytometry (BD FACS Aria III, USA).

### Mitotic inactivation of SH-SY5Y cells

SH-SY5Y cells were treated with 10 μg/ml mitomycin C (Sigma, USA) for 1-4 hours (37°C, 5% CO_2_). Cells were washed (PBS, 3 times) and cultured in a complete culture media (DMEM with 10% FBS, 2mM L-glutamine). Cell proliferation was accessed using xCELLigence Real-Time Cell Analyzer (ACEA BIO, USA).

### Western blot analysis

SH-SY5Y cells and CIMVs were lysed in a RIPA lysis buffer (50mM Tris-HCl pH 7.4, 1% Triton X-100, 0.5% Na-deoxycholate, 0.1% SDS, 150mM NaCl, 2mM EDTA, 1mM PMSF). Protein concentration was determined by bicinchoninic acid (BCA) protein assay (Thermo Scientific, USA). Samples (40μg) were separated in 12% SDS-PAGE under denaturing conditions and then transferred to polyvinylidene fluoride (PVDF) membrane. The membrane was blocked in 5% non-fat dry milk-containing PBS-T (PBS + 0,1% Tween) solution. Proteins were probed using primary antibodies against VEGF (1:1000; SantaCruz Biotech, USA) and β-actin (1:100; GenScript, USA) followed by secondary antibodies (1:5000; GenScript, USA) conjugated with horseradish peroxidase. Protein bands were visualized by ECL reagent (Reagent A: 1.25mM Luminol in 0.1M Tris-HCl pH 8.5; reagent B: 68mM p-Coumaric acid in DMSO; reagent C: 30% hydrogen peroxide) and detected on a ChemiDoc Imaging System by Image Lab software (Bio-Rad, USA). Densitometric analysis, relative to total protein, was performed using Image J software.

### Transmission and scanning electron microscopy

CIMVs were fixed (2.5% glutaraldehyde, 24 hours) and incubated in 1% osmium tetroxide for 1 hour. CIMVs were dehydrated using graded ethanol series, followed by acetone, oxypropylene and epoxy resin embedment. The ultrathin sections were cut using Leica EM ultramicrotome (Leica, USA), mounted on copper grid (Sigma, USA) and contrasted with uranyl acetate and lead citrate (Himmed, Russia). Sections were examined using Jeol SX 1200 electron microscope (Jeol, Japan). For SEM, CIMVs were fixed (10% formalin for 15 min), dehydrated using graded alcohol series and dried at 37°C. Prior to imaging, samples were sputter coated with gold/palladium in a Quorum T150ES sputter coater (Quorum Technologies Ltd, United Kingdom). Slides were analyzed using Merlin (CarlZeiss, Germany) field emission scanning electron microscope. For size determination, CIMVs were obtained in three independent experiments and at least six electron microscope images were done in each experiment for subsequent statistical analysis.

### *In vitro* assessment of capillary-like structure formation by HUVECs

HUVECs (2×10^4^ cells) were seeded in tissue culture wells pre-coated with Matrigel Growth Factor Reduced Basement Membrane Matrix (Becton Dickinson, USA). CIMVs were added to the HUVECs monolayer at a concentration equivalent to 2×10^4^ SH-SY5Y cells based on total protein concentration. HUVECs maintained in MCDB131 media supplemented with 1% FBS was used as a negative control. HUVECs and CIMVs were incubated for 16 hours (37°C, 5% CO_2_) and used to analyze by AxioOberver.Z1 fluorescence microscope (CarlZeiss, Germany). Six replicates were analyzed per experimental group for statistical analyses.

### *In vivo* assessment of pro-angiogenic activity of SH-SY5Y and derived CIMVs

The animal study was approved by KFU ethics committee (protocol #2, date 05.05.2015) according with the rules adopted in KFU and Russian Federal Laws. Angiogenic activity was examined using Wistar rats (Pushchino Laboratory, Russia) with 6 animals per experimental group. Matrigel (200 μl) containing 2x10^6^ SH-SY5Y cells (Group 1), 2x10^6^ mitotically inactivated SHSY5Y cells (Group 2), SH-SY5Y-derived CIMVs at a concentration equivalent to 2×10^6^ SH-SY5Y cells based on total protein concentration (4 mg/ml of total protein) (Group 3) and PBS (Group 4) was injected subcutaneously in the abdominal flanks. SH-SY5 cells and CIMVs were pre-labeled with the membrane dye DiO (Life Technologies, USA). Eight days after injection animals were sacrificed and matrigel plugs collected for examination. Matrigel plugs were frozen in liquid nitrogen and used to make 6 μm sections (NM560Cryo-Star, Thermo Scientific, USA) for immunofluorescent analysis. Also, matrigel plug were paraffin embedded and stained with hematoxylin and eosin. Slides were examined using AxioOberver.Z1 (CarlZeiss, Germany) microscope with Axio Vision 4.8 software (CarlZeiss). For statistical analysis ten hematoxylin and eosin stained slides were analyzed per experimental group.

### Statistical analysis

Statistical analysis was performed using Student’s t-test (Graphpad Software, San Diego, CA) with significance level p < 0.05.

## SUPPLEMENTARY MATERIALS FIGURES


