# Supplementary material for: Cytochalasin B-induced membrane vesicles convey angiogenic activity of parental cells
Source: Oncotarget. 2017 Jul 31;8(41):70496–507. doi: 10.18632/oncotarget.19723 (PMC5642572; doi:10.18632/oncotarget.19723)
Supplement: Supplementary file 1 [file oncotarget-08-70496-s001.pdf]

Cytochalasin B-induced membrane vesicles convey angiogenic activity of parental cells

SUPPLEMENTARY MATERIALS

RESULTS

Influence of CIMVs method production on donor cell viability

We performed cell viability tests by Annexin V/PI and Calcein AM/7-AAD staining to evaluate (I) SH-SY5Y cells viability after 30 min of Cytochalasin B treatment and (II) SH-SY5Y cells viability after 30 sec of vortexing. Staining with Annexin V/ PI and Calcein AM/ 7-AAD indicates cell viability is not impaired following 30 min of Cytochalasin B treatment and after subsequent 30 sec of vortexing (Supplementary Figure 1, 2).

Influence of CIMVs on recipient cell viability

As cytochalasin B binds with actin filaments, trace amounts of cytochalasin B may be retained within CIMVs associated with actin fragments. We therefore evaluated HUVEC viability after addition of SH-SY5Y derived CIMVs using Calcein AM/ 7-AAD staining. SH-SY5Y derived CIMVs were added to the HUVEC culture ($2x10^4$ cells) at a concentration 60 µg/ml (Supplementary Figure 3).

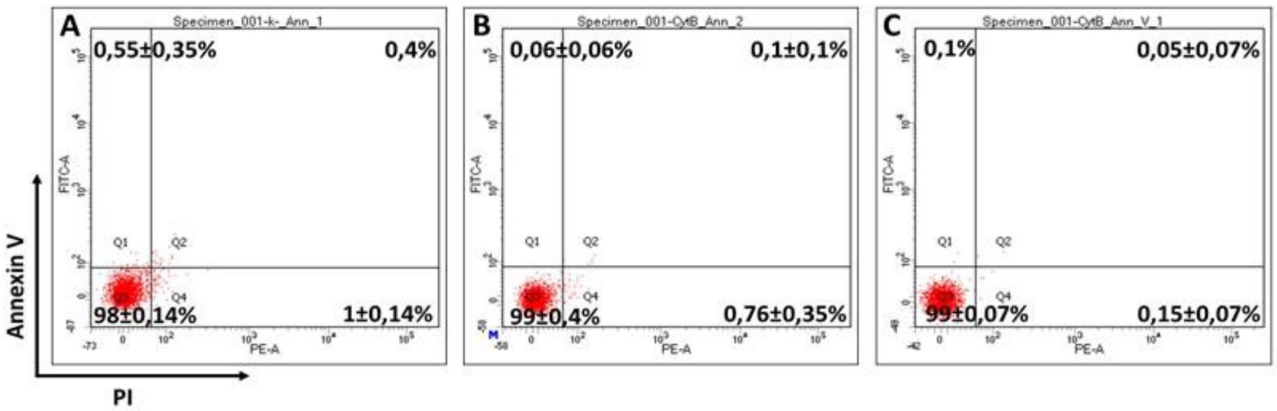

Supplementary Figure 1: Annexin V/ PI staining of: **(A)** - control SH-SY5Y cells; **(B)** - SH-SY5Y cells after 30 min of Cytochalasin B treatment; **(C)** - SH-SY5Y cells after 30 min of Cytochalasin B treatment and subsequent 30 sec of vortexing.

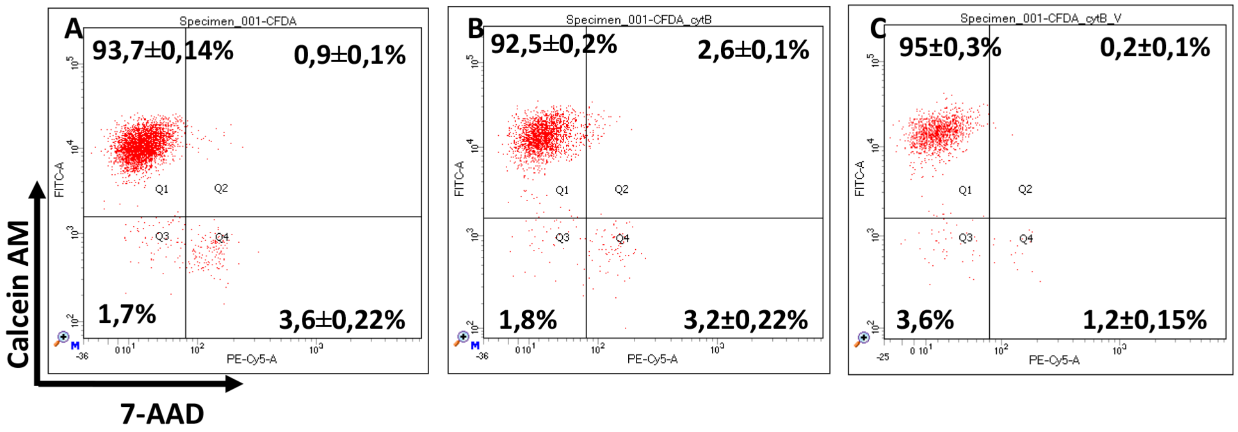

Supplementary Figure 2: Calcein AM/ 7-AAD staining of: **(A)** - control SH-SY5Y cells; **(B)** - SH-SY5Y cells after 30 min of Cytochalasin B treatment; **(C)** - SH-SY5Y cells after 30 min of Cytochalasin B treatment and subsequent 30 sec of vortexing**.**

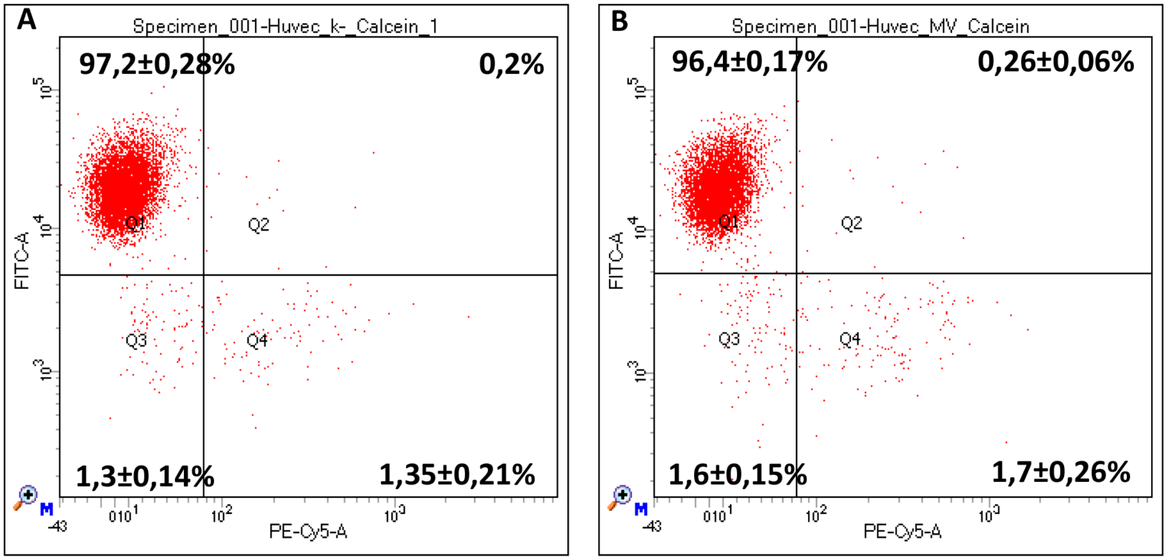

Supplementary Figure 3: Calcein AM/ 7-AAD staining of: **(A)** - control HUVEC; **(B)** - HUVEC after 16 hours of CIMVs SHSY5Y addition**.**
